# Beyond the Apnoea–Hypopnoea Index: Towards Personalised Care of Patients with Suspected Obstructive Sleep Apnoea Through Clinical Phenotyping and Physiological Stratification

**DOI:** 10.3390/jcm15208040

**Published:** 2026-10-16

**Authors:** Mar Mosteiro-Añón, Manuel Casal-Guisande, Alberto Fernández-Villar, María Torres-Durán

**Affiliations:** 1 NeumoVigo I+i Research Group, Galicia Sur Health Research Institute (IIS Galicia Sur), SERGAS-UVIGO, 36312 Vigo, Spain; mar.mosteiro.anon@sergas.es (M.M.-A.); jose.alberto.fernandez.villar@sergas.es (A.F.-V.); maria.luisa.torres.duran@sergas.es (M.T.-D.); 2 Pulmonary Department, Hospital Álvaro Cunqueiro, 36312 Vigo, Spain; 3 School of Industrial Engineering, University of Vigo, 36310 Vigo, Spain; 4 Department of Design in Engineering, University of Vigo, 36208 Vigo, Spain; 5 Centro de Investigación Biomédica en Red, CIBERES ISCIII, 28029 Madrid, Spain; 6 Department of Functional Biology and Health Sciences, University of Vigo, 36310 Vigo, Spain

**Keywords:** hypoxic burden, artificial intelligence, clinical phenotyping, cardiovascular risk stratification, precision medicine, obstructive sleep apnoea

## Abstract

The apnoea–hypopnoea index (AHI) remains central to the diagnosis of obstructive sleep apnoea (OSA), but it incompletely captures both clinical heterogeneity and the physiological burden of respiratory events. We propose a phenotype-guided framework with a common initial clinical assessment followed by two complementary pathways according to the patient’s predominant clinical need: a symptom-oriented pathway based on pre-test clinical phenotyping, and a cardiovascular risk-oriented pathway in which directly measured hypoxic burden (HB), complemented by the respiratory event-related pulse-rate response, may eventually support physiological stratification. In our single-centre cohort of 5385 referrals, three predominantly symptomatic phenotypes accounted for 56.5% of patients and two paucisymptomatic phenotypes (A and E) for 43.5%; these groups had overlapping AHI distributions but different clinical profiles and needs. The reported AUC of 0.87–0.95 reflects supervised reproduction of internally derived cluster labels, not prediction of clinical outcomes or CPAP benefit. HB and pulse-rate response remain candidate physiological markers: evidence for treatment-effect modification derives from post hoc analyses and does not establish validated treatment-allocation criteria. Pre-test phenotyping is sufficiently developed to justify prospective, external, multicentre validation, whereas physiological integration requires methodological standardisation and prospective evaluation before individual treatment decisions can be guided by these markers.

## 1. Introduction

Obstructive sleep apnoea (OSA) is a chronic, heterogeneous disorder whose cardiovascular consequences arise from several interacting acute physiological insults: intermittent nocturnal hypoxaemia, sleep fragmentation and cortical arousals, autonomic activation, and intrathoracic pressure swings [1]. Among these, intermittent hypoxaemia is, in our view, the mechanism most systematically underrepresented by the metric that has governed OSA diagnosis and treatment for decades: the apnoea-hypopnoea index (AHI), which quantifies event frequency but is blind to depth and duration, that is, to the cumulative magnitude of nightly hypoxic stress.

The limitations of the AHI are one plausible, although indirect, contributor to the neutral cardiovascular results of SAVE, RICCADSA, and ISAACC, in which participants were selected largely according to AHI [2,3,4]. Poor CPAP adherence is a well-established complementary explanation: average nightly use in these trials was consistently below the ≥4 h/night threshold associated with measurable cardiovascular benefit in meta-analyses of randomised trials, and the recent secondary analysis of the RICCADSA cohort by Peker et al. [3] found the association between high HB and cardiovascular events was strongest specifically among untreated or non-adherent patients. Other relevant factors include the enrolment of predominantly non-sleepy patients with established cardiovascular disease receiving secondary prevention, exclusion of sleepier patients, and limited follow-up [5,6,7]. The relative contribution of these factors remains uncertain. Accordingly, AHI-based selection should not be interpreted as the sole explanation for the neutral trial results.

In recent years, hypoxic burden (HB), -defined by Azarbarzin et al. as the area under the event-related oxygen desaturation curve, normalised by total sleep time and expressed in %·min·h^−1^ [8,9], has emerged as a candidate biomarker for this blind spot, integrating depth, duration, and frequency of desaturations into a single index. Throughout this article “HB” refers specifically to this originally defined method; modified computational variants (e.g., automatically-derived metrics such as HBOxi) are termed modified HB, and the broader family is termed desaturation area-based metrics, since event definition, sampling window, and baseline method materially affect both the numerical value and cardiovascular associations of these related but non-identical constructs [10,11,12].

In the post hoc ISAACC re-analysis, Pinilla et al. reported a formal treatment-by-modified-HB interaction for cardiovascular outcomes (*p* = 0.023; adjusted *p* = 0.035) [13]. Among patients above the cohort median (73.1%·min·h^−1^), the unadjusted estimate favoured CPAP (HR 0.57, 95% CI 0.34–0.96), whereas the adjusted estimate crossed unity (HR 0.61, 95% CI 0.36–1.03); below the median, the estimate was HR 1.33 (95% CI 0.79–2.25), but a wide interval compatible with potential benefit, no effect, or potential harm from treatment. This post hoc interaction supports modified HB as a candidate predictive biomarker, but not as a validated treatment-allocation criterion. We therefore distinguish a prognostic marker associated with cardiovascular risk from a predictive marker demonstrating treatment-effect modification, and from a clinical allocation criterion, which would require prospective confirmation under a prespecified protocol.

Artificial intelligence (AI) may support, but is not synonymous with, this stratification [14,15]. We propose a phenotype-guided framework beginning with a common initial clinical assessment and followed by two complementary pathways (see Table 1). Pathway 1 uses pre-test clinical-demographic phenotyping to identify clinically interpretable profiles and potential diagnostic needs. Pathway 2 addresses cardiovascular risk-oriented physiological stratification using HB and the respiratory event-related pulse-rate response measured from the patient’s sleep recording. Automated extraction of physiological metrics is signal processing rather than AI; machine learning would become relevant if these measured variables were subsequently integrated with clinical data to predict outcomes or treatment response. Unsupervised phenotype discovery, supervised assignment to those phenotypes, and future prediction of clinical outcomes or treatment response are distinct tasks and require separate validation.

## 2. Pathway 1: Pre-Test Clinical Phenotyping and Symptom-Oriented Evaluation

This pathway is grounded in our cohort of 5385 consecutive patients referred with suspected OSA at a single centre, in whom unsupervised k-prototypes clustering of pre-test clinical-demographic variables identified five clinically interpretable phenotypes [16]. Three predominantly symptomatic phenotypes (B, C, and D) accounted for 56.5% of referrals, whereas phenotypes A and E were predominantly paucisymptomatic and accounted for 43.5%. Importantly, AHI distributions overlapped across phenotypes; the clusters should therefore be interpreted as exploratory profiles of differing clinical needs rather than as discrete categories of OSA severity or treatment response.

Phenotypes A and E are particularly relevant to the cardiovascular-risk question because they were predominantly paucisymptomatic. Phenotype A comprised middle-aged men with few symptoms and relatively low comorbidity, whereas phenotype E comprised older, mostly retired men with the highest prevalence of hypertension (36.2%) and diabetes (9.8%) and the highest mean AHI (32.6 events/h). HB was not measured in this cohort. We therefore do not infer that either phenotype has elevated HB or that symptoms underestimate its hypoxic load. Rather, these paucisymptomatic phenotypes identify clinically relevant populations in whom the expected purpose of CPAP may be less symptomatic and in whom prospective physiological stratification warrants study.

The symptomatic phenotypes illustrate a different clinical need. Phenotype B, predominantly women, showed substantial non-restorative sleep and fatigue despite the lowest mean AHI; phenotypes C and D were characterised by marked sleepiness and sleep fragmentation, respectively [16]. These profiles support a broader symptom-oriented evaluation rather than assuming that symptom burden is explained by AHI alone.

These findings support the feasibility and potential usefulness of pre-test clinical phenotyping based on variables available before the sleep study. The decision-support system, a Random Forest classifier developed after unsupervised phenotype identification and recursive feature elimination, achieved an AUC of 0.87–0.95 in an internal test set using ten variables (age, Epworth score, BMI, neck circumference, perceived non-restorative sleep, perceived sleepiness, absence of morning grogginess, absence of concentration difficulties, occupational status, and female sex). This performance reflects the model’s ability to assign patients to cluster labels derived within the same single-centre cohort (80%/20% train/test split), rather than to predict OSA, cardiovascular events, CPAP response, or clinical benefit. We have not demonstrated superiority over STOP-Bang, NoSAS, conventional statistical models, or simpler clinical categories.

The approach should therefore be considered sufficiently developed to justify prospective, external, multicentre validation, rather than ready for clinical implementation. Such validation should include assessment of cluster stability, calibration and discrimination, geographical and temporal transportability, subgroup performance, comparison with simpler clinical approaches, management of missing data and dataset shift, and prospective evaluation of clinical utility and workflow impact. Within the proposed framework, pre-test phenotyping is intended to support clinical judgement by helping to identify the patient’s predominant clinical profile and subsequent evaluation within the symptom-oriented or cardiovascular risk-oriented pathway and should not be used to deny or delay access to sleep testing.

## 3. Pathway 2: Cardiovascular Risk-Oriented Physiological Stratification

For predominantly paucisymptomatic patients, particularly phenotypes A and E, the relevant uncertainty is whether OSA-related physiology identifies cardiovascular risk and, ultimately, differential cardiovascular benefit from CPAP.

HB should be calculated directly from oximetric or polysomnographic signals after the sleep study; automated extraction is physiological signal processing, not AI. Machine learning would become relevant only if measured HB and other physiological variables were integrated with clinical data to estimate outcomes or treatment response. This pathway remains investigational and is not currently a treatment-allocation algorithm.

Post hoc analyses provide hypothesis-generating evidence of treatment-effect heterogeneity. In ISAACC, modified HB showed a formal treatment-by-HB interaction with CPAP [13]. In RICCADSA, the respiratory event-related pulse-rate response similarly showed a significant treatment-by-marker interaction. In the pooled analysis of RICCADSA, ISAACC, and SAVE [17], high-risk OSA was defined as HB > 87.1%·min·h^−1^ or a pulse-rate response > 9.4 beats/min, corresponding to upper-tertile distributional cut-offs rather than validated clinical thresholds. The treatment-by-risk interaction was significant, although the analysis remained post hoc with respect to the original trials [17]. These findings support HB and the respiratory event-related pulse-rate response as candidate predictive biomarkers, but not as validated criteria for CPAP treatment allocation.

The values 60.7%·min·h^−1^ (RICCADSA), 73.1%·min·h^−1^ (ISAACC), and 87.1%·min·h^−1^ (pooled multi-trial analysis) arise from different cohorts, analytical contexts, and, in some cases, different implementations of desaturation-area metrics [7,13,17]. They should not be interpreted as interchangeable or universal clinical thresholds. Their heterogeneity reinforces the need for algorithm-specific reporting, external validation, and prospective testing before any threshold is used for treatment allocation. CPAP addresses mechanical upper-airway obstruction and its downstream physiological consequences, including intermittent hypoxaemia. Whether HB, as a physiological measure complementary to the AHI, can improve the identification of patients more likely to derive cardiovascular benefit from CPAP remains a hypothesis supported by post hoc analyses and requires prospective validation before it can be used as a treatment-allocation criterion.

In both contexts, HB, -measured directly or via modified variants-, faces a technical obstacle: lack of algorithmic standardisation across studies.

## 4. The Unresolved Problem: HB Standardisation and Its Implications for AI

We reserve HB for Azarbarzin et al.’s original method: event-related desaturation area normalised by total sleep time, using a patient-specific search window and pre-event baseline [8,9]. Other area-based measures, -HBOxi, REDTA, and related automatically-derived metrics-, assess related but non-identical constructs and should be called modified HB or, collectively, desaturation area-based metrics, rather than grouped indiscriminately under “HB”. This matters because even among related metrics, results depend critically on technical choices that vary across studies: the SpO_2_ baseline method (event-specific, recording-specific, or fixed), the desaturation search window, detection threshold (≥3% vs. ≥4%), and oximeter type [9,10,11,12]. He et al. showed baseline method choice substantially alters HB values and their mortality association [10]; De Chazal et al. extended this to automatically identified desaturations with similar conclusions [11]; a recent comparison of three area-based algorithms confirmed event definition, sampling window, and baseline selection materially affect both values and cardiovascular associations [12]. This does not mean HB itself lacks definition, -the original method is well specified-, but that the broader family of desaturation area-based metrics requires precise naming and algorithm-specific reporting in every study.

This heterogeneity has direct consequences for AI: a model trained on modified HB variants or device-specific data learns a relationship tied to that pipeline, with unknown generalisability to other centres, algorithms, or oximeters in the absence of a consensual standard. Terminological confusion compounds this: HB, Tc90, and ODI are sometimes used as equivalent when they represent distinct constructs [18]: models trained on Tc90 (% sleep time with SpO_2_ < 90%) do not predict the same construct as strict HB, nor as ODI (3% desaturation criterion).

A relevant step in the right direction is the development of HBOxi, calculating modified HB from automatically identified oximetric desaturations without manual event scoring [19], showing Spearman correlation 0.81 with manually calculated HB in SHHS and similar mortality prediction (adjusted HR 1.15 per 1 SD). This automation is a prerequisite for integrating measured HB into AI pipelines for home recordings and ambulatory polygraphy—the devices accessible in overburdened real-world sleep units.

No single HB threshold currently has broad external validation: values reported in RICCADSA, ISAACC, and the pooled multi-trial analysis are cohort- or context-specific [7,13,17]. Because HB is a continuous physiological measure and its association with cardiovascular risk appears continuous, it should preferentially be modelled continuously, allowing potential non-linear effects; clinically useful thresholds should subsequently be evaluated using appropriate decision-analytic methods and prospectively validated. At minimum, HB studies should report the respiratory-event definition; oximeter characteristics and sampling frequency; baseline definition; event-related search window; desaturation threshold; artefact and missing-data handling; normalisation by sleep time or recording time; algorithm/software version; and reproducibility across devices and laboratories. Original HB, modified HB/HBOxi, REDTA, Tc90, and ODI should be named separately because they represent related but non-identical constructs [8,9,10,11,12,17,18].

## 5. An Open Question with Implications for Model Design

An important question that current AI, and HB considered as a single summary metric, cannot resolve is whether two patients with the same HB but different desaturation profiles experience equivalent biological stress.

Experimental studies suggest that the depth and temporal pattern of intermittent hypoxia may modulate inflammatory, oxidative, and sympathetic responses [1,20,21]. Epidemiological evidence is less conclusive: in the Osteoporotic Fractures in Men Study, re-weighting desaturations to emphasise or attenuate depth did not materially alter the association between HB and cardiovascular mortality [8]. The clinical relevance of desaturation shape therefore remains uncertain and requires prospective validation).

This has direct design implications: a model predicting HB as a single scale cannot discriminate these patterns. Future work should target disaggregated HB components, -mean desaturation depth, mean event duration, rate of SpO_2_ fall-, for finer pathophysiological resolution. Until such disaggregation is validated in prospective studies with major clinical events, HB as a single target remains, in our assessment, a more informative starting point than AHI, but understood as an approximation rather than a complete description of the phenomenon.

## 6. An Operational Perspective: What AI Can and Cannot Do Today

With appropriate caution, what AI can currently offer in OSA stratification and what it cannot yet deliver.

What it can currently do: assign patients to internally derived clinical phenotypes using pre-test variables, with AUC 0.87–0.95 in a single-centre internal test set [16]. This performance concerns reproduction of cluster labels, not prediction of outcomes. The potential value of unsupervised phenotyping is the identification of multidimensional clinical profiles that may not be captured by a single probability score, but added value over STOP-Bang, NoSAS, simpler statistical models, or conventional clinical categories has not yet been demonstrated.

What it cannot yet do: determine whether CPAP will prevent a cardiovascular event in an individual patient or use HB as a validated treatment-allocation criterion. HB should be measured from the physiological recording rather than predicted from pre-test clinical variables as a surrogate. Prospective AI evaluation should include geographical and temporal external validation, calibration, comparison with clinical standards and simpler models, subgroup performance by sex, age, ethnicity, centre, and device type, explicit management of missing data and dataset shift, clinical-utility and workflow-impact analyses, transparency and interpretability, and post-implementation monitoring.

Neither pathway is currently validated for individual clinical decision-making. Pathway 1 is sufficiently developed to justify prospective, external, multicentre validation. Pathway 2 requires methodological standardisation of HB, device and ambulatory validation, and prospective trials in which physiological markers and treatment-by-marker interactions are prespecified. The framework should therefore be regarded as a set of testable hypotheses rather than an established clinical algorithm.

## 7. Conclusions

The AHI remains the diagnostic anchor of OSA and retains important descriptive value, but its isolated use is insufficient to capture the clinical heterogeneity of referred patients or to identify differential cardiovascular benefit from CPAP. The distinction between predominantly symptomatic phenotypes and the paucisymptomatic phenotypes A and E provides the clinical rationale for two complementary pathways: symptom-oriented evaluation and cardiovascular risk-oriented physiological stratification. HB is not a replacement for AHI; it is a candidate physiological complement whose prognostic value is supported by observational data and whose predictive role for CPAP benefit remains to be prospectively validated.

AI may support this framework, but its role should be defined precisely. Pre-test phenotyping is sufficiently developed to justify prospective, external, multicentre validation, whereas the integration of measured physiological markers with clinical information remains to be developed and prospectively evaluated. Standardising HB and prospectively testing whether HB and the respiratory event-related pulse-rate response modify cardiovascular benefit from CPAP are necessary before these markers can be used for treatment allocation.

## Figures and Tables

**Table 1:** Proposed phenotype-guided framework integrating symptom-oriented and cardiovascular risk-oriented pathways in patients referred with suspected obstructive sleep apnoea.

	Pathway 1: Symptom-Oriented Clinical Phenotyping	Pathway 2: Cardiovascular Risk-Oriented Physiological Stratification
Objective	Characterise pre-test clinical phenotypes and identify the patient’s predominant clinical profile and needs.	Characterise OSA-related physiological burden relevant to cardiovascular risk and investigate heterogeneity in cardiovascular benefit from CPAP.
Data source	Clinical and demographic variables available at the first clinical assessment, before sleep testing.	Physiological signals obtained from the sleep recording, including directly measured HB and the respiratory event-related pulse-rate response, considered alongside AHI and clinical information.
Clinical context/candidate population	Patients referred with suspected OSA, particularly those with a predominantly symptomatic presentation requiring assessment of the factors contributing to impaired sleep and daytime symptoms.	Predominantly paucisymptomatic patients in whom cardiovascular risk is a major clinical concern. Phenotypes A and E represent candidate populations for prospective evaluation, but elevated HB has not been demonstrated in these phenotypes.
Proposed clinical role	Support clinical judgement by characterising the patient’s predominant symptomatic profile and informing subsequent evaluation; not intended to restrict or delay access to sleep testing.	Complement AHI-based assessment with physiological markers that may improve cardiovascular risk stratification and, potentially, identify heterogeneity in cardiovascular benefit from CPAP.
Current evidence	Unsupervised clustering identified five clinically interpretable phenotypes in a single-centre cohort of 5385 patients. A subsequent supervised Random Forest classifier achieved an AUC of 0.87–0.95 for assigning patients to the internally derived cluster labels using ten pre-test variables.	HB has shown prognostic associations with cardiovascular outcomes. Post hoc analyses of randomised trials suggest treatment-effect modification by HB and/or the respiratory event-related pulse-rate response, whereas comparable effect modification has not been demonstrated using AHI-based stratification.
Main limitations	Single-centre data; AUC reflects reproduction of internally derived cluster labels rather than prediction of clinical outcomes, cardiovascular events, or CPAP response. Added value over simpler clinical approaches has not been demonstrated.	Lack of standardisation across HB implementations and devices; absence of a universally validated clinical threshold; evidence for treatment-effect modification derives from post hoc analyses and does not establish a validated treatment-allocation criterion.
Next validation step	Prospective geographical and temporal external validation, including calibration, cluster stability, subgroup performance, comparison with simpler clinical approaches, management of missing data and dataset shift, and evaluation of clinical utility and workflow impact.	Standardisation and external validation of physiological measurements, followed by prospective studies with prespecified treatment-by-marker interactions to determine whether HB and the respiratory event-related pulse-rate response provide incremental value for cardiovascular treatment allocation.

## Data Availability

No new data were created or analyzed in this study. Data sharing is not applicable to this article.
